# Baby, You Light-Up My Face: Culture-General Physiological Responses to Infants and Culture-Specific Cognitive Judgements of Adults

**DOI:** 10.1371/journal.pone.0106705

**Published:** 2014-10-29

**Authors:** Gianluca Esposito, Jun Nakazawa, Shota Ogawa, Rita Stival, Akiko Kawashima, Diane L. Putnick, Marc H. Bornstein

**Affiliations:** 1 Department of Psychology and Cognitive Science, University of Trento, Rovereto, TN, Italy; 2 Division of Psychology, School of Humanities and Social Sciences, Nanyang Technological University, Singapore, Singapore; 3 Department of Developmental Science, Faculty of Education, Chiba University, Chiba, Japan; 4 United Graduate School of Education, Tokyo Gakugei University, Chiba, Japan; 5 School of Social Welfare, Tokyo University of Social Welfare, Tokyo, Japan; 6 Eunice Kennedy Shriver National Institute of Child Health and Human Development, National Institutes of Health, Bethesda, Maryland, United States of America; Birkbeck, University of London, United Kingdom

## Abstract

Infants universally elicit in adults a set of solicitous behaviors that are evolutionarily important for the survival of the species. However, exposure, experience, and prejudice appear to govern adults' social choice and ingroup attitudes towards other adults. In the current study, physiological arousal and behavioral judgments were assessed while adults processed unfamiliar infant and adult faces of ingroup vs. outgroup members in two contrasting cultures, Japan and Italy. Physiological arousal was investigated using the novel technique of infrared thermography and behavioral judgments using ratings. We uncovered a dissociation between physiological and behavioral responses. At the physiological level, both Japanese and Italian adults showed significant activation (increase of facial temperature) for both ingroup and outgroup infant faces. At the behavioral level, both Japanese and Italian adults showed significant preferences for ingroup adults. Arousal responses to infants appear to be mediated by the autonomic nervous system and are not dependent on direct caregiving exposure, but behavioral responses appear to be mediated by higher-order cognitive processing based on social acceptance and cultural exposure.

## Introduction

The ethologist Konrad Lorenz [1], [2] famously identified a set of physiognomic features in infants known as *kindchenschema* that effectively elicit affection and nurturance from adults. These facial characteristics include a relatively large round head, big eyes, small nose and mouth, and chubby cheeks. In Lorenz's formulation, baby schema release a set of parental care behaviors in conspecifics and are evolutionarily vital for survival (see [3], [4]). Moreover, images with more pronounced baby schema elicit stronger self-reported motivation for caregiving compared with unmanipulated and low baby schema images [5]; higher baby schema images also activate brain regions (e.g., the nucleus accumbens) that implicate potential engagement of the mesolimbic system [6]. Using fMRI, Caria et al. [7] observed stronger activation in thalamocingulate, anterior insula, and the supplementary motor area and premotor cortex to unknown infant faces compared with carefully matched adult and nonhuman infant and adult faces. Activation of brain areas involved in motor preparation in response to infant faces was interpreted to reflect implicit preparation to interact with infants. Glocker et al. and Caria et al. recorded similar response patterns in parents and in non-parents, suggesting the generalized power of *kindchenschema* to activate neural systems involved in empathy and caregiving [8]. Sensitivity to Lorenz's baby schema can be found in children [9] and even in infants as young as 4 months of age themselves [10]. This generalized pattern speaks to the universal efficacy of infant-like appearance in eliciting alloparental care and may be at a premium in humans whose offspring cannot independently self-sustain but must rely for years on adult care for their survival and well-being.

Attention captured by *kindschenschema* is immediate, automatic, and unconscious. Using MEG, Kringelbach et al. [11] found that observing unfamiliar infant faces is associated with activity in the orbitofrontal cortex (OFC) and enhanced responsiveness in the fusiform face area that is not present when observing adult faces. Moreover, OFC activation has very rapid onset (∼130 ms), which the authors interpreted as unconsciously directing attention to the infant face. The idea of implicit activation to infants versus adults was further confirmed by Senese and colleagues [12] who assessed unconscious and unique positively valenced associations to infant faces using a Single-Category Implicit Association Test. Together, these results support the idea that human infant faces represent highly biologically relevant stimuli that immediately and automatically capture attention and are implicitly associated with positive emotions.

The same is not true of adult faces. It is widely acknowledged that for many types of stimuli, including faces, exposure increases implicit ingroup attitudes (in both adults and children [13]) and attraction even when the exposure is unconscious [14]–[15]. Experimental research points to discrimination that favors one's own group [16]. Furthermore, bias appears to be enhanced by increased favoritism toward ingroup members rather than by increased hostility toward outgroup members [17]. Several reasons have been offered for ingroup preferences. Zajonc [16], [18], for example, suggested that ingroup preferences may be based on mere exposure which renders an object or situation more positive and attractive. Repeated exposure allows the individual to readily distinguish between objects or situations that are safe from those that are not, and may thereby constitute a basis of social attachment and social organization. As individuals are continually exposed to specific objects or situations, they soon come to prefer them. In consequence of continued exposure, the faces of people in the ingroup are preferred to faces from the outgroup [13]. To date, the majority of evidence for this preference has come from the behavioral social psychology literature.

Because previous theorizing and research have shown that people express universal preference for infant faces but ingroup preferences for adult faces, we hypothesized (H1) that both Japanese and Italian adults, although belonging to contrasting cultures, would show similar patterns of results for both the physiological and behavioral responses to infant faces and (H2) both groups would dissociate physiological and behavioral responses; (H3) infant faces as opposed to adult faces would elicit greater physiological activation (measured as an increase in facial temperature) independent of the infant ethnicity (stimulus ethnicity); and (H4) participants from both cultures would show greater behavioral preference (measured as explicit assessment of willingness to interact) to ingroup adults (same ethnicity) vs. outgroup adults.

To begin to address these hypotheses, we conducted two parallel studies in two contrasting cultures, Japan and Italy, where we investigated physiological and behavioral responses of adults to unfamiliar Asian and Caucasian infant and adult faces. Preference for the ingroup is practically an anthropological and social psychological universal. However, ingroup preference may be driven by different motives and take different forms. For example, ingroup preference would be expected to relate to openness in interactions with other individuals from the same group in self-oriented cultures (as Western societies are thought to be), whereas maintaining good relationships and face-saving with people of the same group are socially desirable in group-oriented cultures (as Eastern societies are thought to be; see for example [19]). Thus, considering that cultural values greatly influence interpersonal and group relationships [20]–[23] as well as why people from the same group are approached and favored in different cultures, we recruited a Japanese sample as representative of an Eastern group-oriented society and contrasted it with an Italian sample as representative of a Western self-oriented society. We selected non-parent female participants because we wanted to avoid effects of previous parenting experience with infants and we wished to test the generality of preferences outside parenthood status. To investigate physiological responses, we used a novel, non-contact, non-invasive method for tracking emotional change, infrared thermography, and we measured facial skin temperature. Infrared thermography has been successfully used to measure facial skin temperature as a sign of physiological arousal. Increases in skin temperature are generally associated with greater arousal [18], [24]–[26] and greater activation (higher levels of oxygenation of hemoglobin [27]). This technology has also been used in different cultural groups (e.g., in the U.S.A. [26]; in Japan [25], 1990; in Italy [28]) as well as in different age groups (in infants [29]; in adults [30]).

## Methods

### Participants

Exclusion criteria common to both studies were parenthood, pregnancy, having an infant in the same household, employment or engaging in volunteer activities that involve infants, and neurological or psychiatric disorders, including substance abuse/dependence and psychotropic medication. The study was approved by the Internal Ethical Committee of the Chiba University, and was conducted according to the principles expressed in the Declaration of Helsinki. Written informed consent was obtained from the participants.

### Visual stimuli

A total of 20 color pictures of infant and adult human female faces either Asian (Japanese) or Caucasian (Central Europe), 5 for each category, were equated for brightness and color-balance using Adobe Photoshop 9.0.1 (the average brightness value of all pixels fell between 125 and 220 cd/m^2^). All pictures showed a frontally oriented, neutrally expressive face on a white background; head size was matched across stimuli. Face stimuli came from public domain databases [31]–[33] or were images taken by a professional photographer and edited by a private graphics company (Fulvia Riccardi). As required by Chiba University Ethical guidelines, written informed consent was obtained from legal guardians (mother) on behalf of the photographed minors. To exclude any potential influence of differential attractiveness [3], [34], pictures were selected within a larger database (*n* = 96 with the same characteristics and sources), presented on a laptop (for 5 s each) interleaved in one of two random orders, and rated by 50 adults (Japanese and Italian females, *M* age = 25.05, *SD* = 2.15) on a 7-point Likert scale ranging from *unattractive* to *attractive*. The 20 stimuli selected for the experiment were rated as moderately attractive (*M* = 3.1, *SD* = 1.2).

### Stimulus presentation

After stimulus preparation, the 20 picture files were presented to seated participants on a ∼48-cm screen (for 5 s each) placed at a distance of 50 cm in one of two random orders. Adult and infant pictures alternated with face ethnicity intermixed. The 5-s presentations were interspersed with 10 s of a neutral screen. Pilot work showed that increases and decreases in facial temperature reached a peak after 5 s and that 10 s of a neutral screen was adequate to allow face temperature to return to baseline.

### Measurement devices and environment

**Figure 1:**
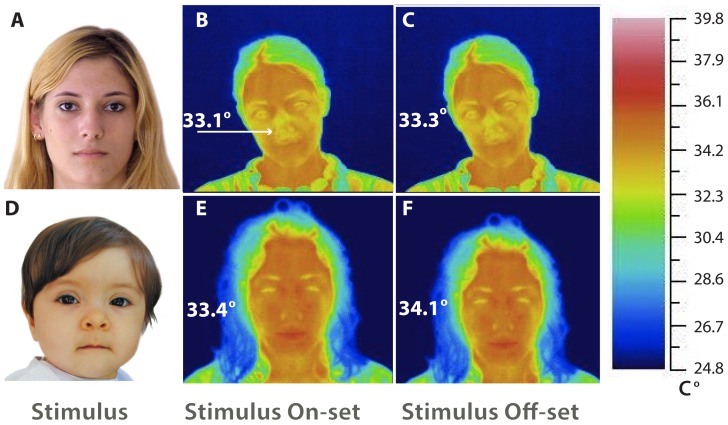
Examples of responses to the presentation of faces of Caucasian adults (A–C) and Caucasian infants (D–F). In the A–C sequence, A is the presented stimulus (Caucasian adult) and B and C show tip-of-the-nose facial temperatures, respectively, at the onset (33.1°C) and at the offset (33.3°C) of picture presentation. The white arrow in B points to the tip of the nose. In the D–F sequence, D is the presented stimulus (Caucasian infant), and E and F show the facial temperatures, respectively, at the onset (33.4°C) and at the offset (34.1°C) of picture presentation.

A thermal imaging system (Avio TVS-200EX) and its software (Avio thermography studio 2007, version 4.8) were used to measure facial skin temperature. The testing environment was an artificial climate chamber maintained at a constant 25°C and 55% humidity. The thermocamera was placed 1 m in front of the participant and was set to measure the participant's overall face skin temperature. The participant's skin temperature of the whole face (from the neck up) was recorded as a thermal image taken at the onset and offset of the presentation of each visual stimulus (see Fig. 1 and Table 1). Other studies in the literature have used different points of the face (cheeks and forehead) to measure facial temperature [26]. Our pilot study revealed that for our class of stimuli, the pattern of changes in facial temperature was homogeneous, and thus the tip of the nose was chosen as a focal variable because it is a point on the face that is constant and easy to target and detect reliably across all participants. A video camera, placed next to the thermocamera, was used to record overall behavior and verify that participants were looking at the stimulus faces.

**Table 1:** Means and Standard Deviations of the tip of the nose temperature at onset (on) and offset (off) of picture presentation.

	Japan				Italy			
	Onset		Offset		Onset		Offset	
	*mean*	*sd*	*mean*	*sd*	*mean*	*sd*	*mean*	*sd*
***Asian***								
***Adults***	32.06	2.88	32.11	2.80	32.13	2.30	32.19	2.27
***Infants***	32.05	2.85	34.03	1.59	32.36	2.24	33.30	2.26
***Caucasian***								
***Adults***	32.22	2.39	32.33	2.40	32.23	2.20	32.36	2.23
***Infants***	32.38	2.32	33.86	1.99	32.40	2.19	33.36	2.18

### Behavioral data

Immediately after thermoimaging, participants rated their feelings while re-viewing the 20 faces. Feelings were rated on three 7-point scales: willingness to approach the person, willingness to smile at the person, and willingness to communicate with the person. All scales ranged from *not at all* to *extremely* and were selected on the basis of the literature on adult–infant interactions to assess the degree of adults' typical and prominent responses to faces [7], [35]–[38]. The three items were highly correlated (*rs* range = .75–.77) and so were averaged to construct a single index of Willingness to Interact with the person (Cronbach's α = .91).

### Preliminary analyses and analytic plan

Two independent variables represented stimulus characteristics: age (infants vs. adults) and ethnicity (Asian vs. Caucasian). The two dependent variables were: tip of the nose temperature (measured at the onset and offset of stimulus presentation) and ratings for Willingness to Interact with the person. Prior to data analysis, univariate and multivariate distributions of all the variables were examined for normality, homogeneity of variance, outliers, and influential cases in each study. All variables were normally distributed. The distance of each case to the centroid was evaluated to screen for multidimensional outliers. The dependent variables were uncorrelated at stimulus onset (*r* = −.05, *ns*) and stimulus offset (*r* = .03, *ns*).

To determine which feature(s) of the stimuli were operative, we employed repeated-measures ANOVA (RM-ANOVA) with (i) tip of the nose temperature as the dependent variable, two between factors (stimulus age and ethnicity) and two repeated factors (time of presentation, i.e., onset vs. offset, and 20 trials, i.e., individual faces) and (ii) Willingness to Interact with the person as the dependent variable with two between factors (age and ethnicity) and one repeated factor (20 trials, i.e., individual faces).

We used the same experimental procedures and analytic plan in two parallel studies conducted in two urban areas in two contrasting countries: Chiba in Japan and Trento in Italy.

### Study 1: Experiment in Japan – Participants

A total of 33 Japanese females (*M* age = 21.1 years±.8) recruited in the urban area of Chiba, Japan, participated.

### Study 1: Experiment in Japan – Results

#### Facial temperature

**Figure 2:**
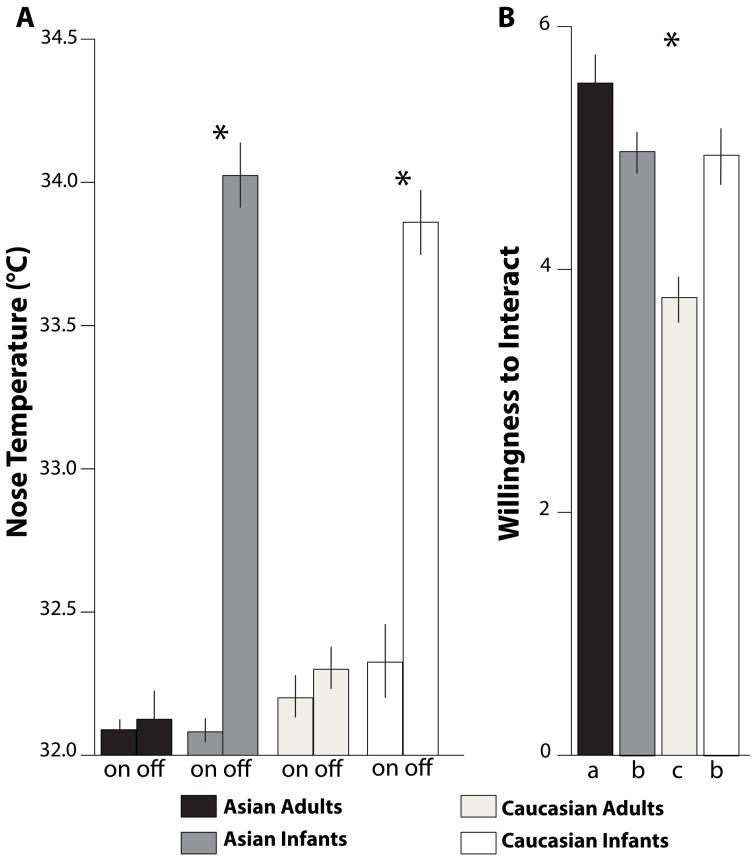
Japanese participants. Responses to the presentation of the faces of Asian adults (black), Asian infants (dark grey), Caucasian adults (light grey), and Caucasian infants (white). (A) Mean levels (± SEM) of the tip of the nose temperature at onset (on) and offset (off) of picture presentation. (B) Mean levels (± SEM) of scores for Willingness to Interact with the person. Bars with different subscripts (a, b, and c) are significantly different in Tukey HSD post-hoc tests.

Mean (and *SD*) levels of the temperature (°C) at stimulus onset and offset (see Fig. 1) are shown in Fig. 2A and given in Table 1. The RM-ANOVA showed significant main effects for time of presentation, *F*(1,1163) = 134.27, *p*≤.001, η_p_^2^ = .10 (onset<offset), and age, *F*(1,1163) = 141.99, *p*≤.001, η_p_^2^ = .11 (infants>adults), which were subsumed by a significant interaction for Time of presentation *X* Age, *F*(1,1163) = 108.76, *p*≤.001, η_p_^2^ = .09. Infant faces (both Asian and Caucasian) elicited warmer face temperatures than adult faces at the offset of the picture presentation (*M* difference _(Infant Faces – Adult Faces)_ = 1.81, SE = .009, *p*<.01) but not at the onset (*M* difference _(Infant Faces – Adult Faces)_ = −.11, SE = .009, *ns*). Neither a main effect for stimulus ethnicity nor any other interaction effects emerged.

#### Willingness to interact

Fig. 2B and Table 2 show the scores for Willingness to Interact. The RM-ANOVA showed main effects for age, *F*(1,642) = 87.77, *p*≤.001, η_p_^2^ = .06, and for ethnicity, *F*(1,642) = 174.99, *p*≤.001, η_p_^2^ = .12. These main effects were also subsumed by a significant interaction, *F*(1,642) = 180.60, *p*≤.001, η_p_^2^ = .12. Tukey HSD post-hoc analyses with Holm adjustment showed that Japanese participants expressed the highest Willingness to Interact with pictures of Asian (Japanese) adults and it differed statistically from their Willingness to Interact with Asian infants (*M* difference _(Asian Adults – Asian Infants)_ = .23, *SE* = .005, *p*≤.05), Caucasian infants (*M* difference _(Asian Adults – Caucasian Infants)_ = .22, *SE* = .005, *p*≤.05), and Caucasian adults (*M* difference _(Asian Adults – Caucasian Adults)_ = .53, *SE* = .006, *p*≤.001). The lowest Willingness to Interact which Japanese participants expressed was for Caucasian adults, significantly lower than Asian infants (*M* difference _(Caucasian Adults – Asian Infants)_ = −.30, *SE* = .003, *p*≤.05) and Caucasian infants (*M* difference _(Caucasian Adults – Caucasian Infants)_ = −.31, *SE* = .005, *p*≤.05). No difference emerged for Willingness to Interact with infants (*M* difference_ (Caucasian Infants – Asian Infants)_ = −.01, *SE* = .002, *ns*).

**Table 2:** Means and Standard Deviations of the Willingness to Interact.

	Japan		Italy	
	*mean*	*sd*	*mean*	*sd*
***Asian***				
***Adults***	5.42	0.96	3.64	1.53
***Infants***	5.19	1.02	5.13	1.41
***Caucasian***				
***Adults***	3.89	1.39	5.35	1.19
***Infants***	5.20	1.08	5.14	1.15

### Study 2: Experiment in Italy – Participants

A total of 32 Italian females (*M* age = 24.6 years±2.2) recruited in the urban area of Trento, Italy, participated.

### Study 2: Experiment in Italy – Results

#### Facial temperature

**Figure 3:**
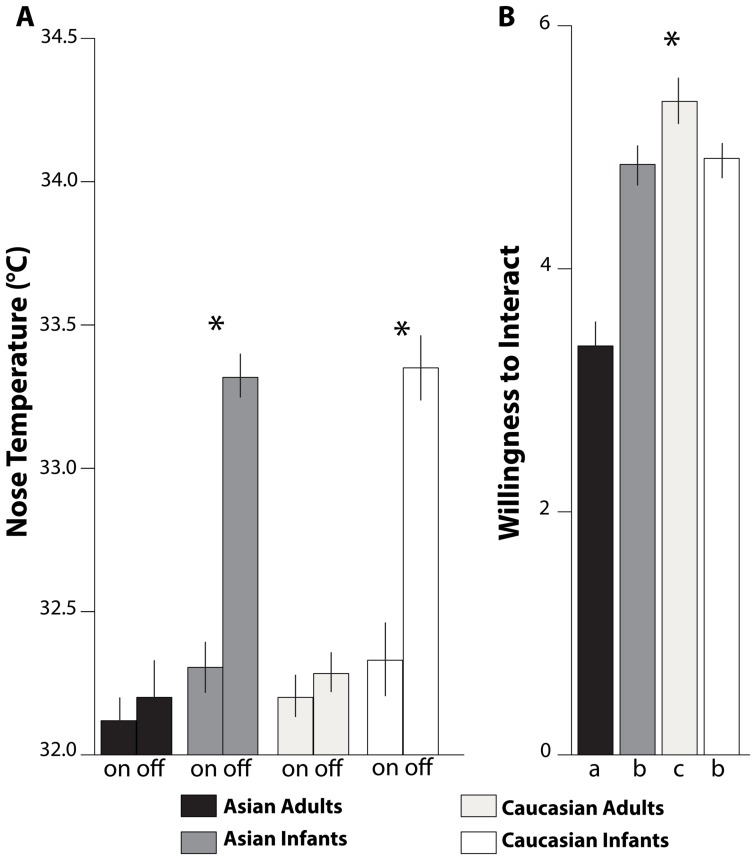
Italian participants. Responses to the presentation of the faces of Asian adults (black), Asian infants (dark grey), Caucasian adults (light grey), and Caucasian infants (white). (A) Mean levels (± SEM) of the tip of the nose temperature at onset (on) and offset (off) of the picture presentation. (B) Mean levels (± SEM) of scores for Willingness to Interact with the person. Bars with different subscripts (a, b, and c) are significantly different in Tukey HSD post-hoc tests.

Mean (and *SD*) levels of the temperature (°C) at stimulus onset and offset are shown in Fig. 3A and given in Table 1. The RM-ANOVA showed significant main effects for time of presentation, *F*(1,1241) = 111.59, *p*≤.001, η_p_^2^ = .08 (onset<offset), and age, *F*(1,1241) = 160.20, *p*≤.001, η_p_^2^ = .11 (infants>adults), which were subsumed by a significant interaction for Time of presentation *X* Age, *F*(1,1241) = 74.23, *p*≤.001, η_p_^2^ = .06. Infant faces (both Asian and Caucasian) elicited warmer face temperatures than adult faces at the offset of picture presentation (*M* difference _(Infant Faces – Adult Faces)_ = 1.05, SE = .008, *p*<.01) but not at the onset (*M* difference _(Infant Faces – Adult Faces)_ = .02, SE = .007, *ns*). Neither a main effect for stimulus ethnicity nor any other interactions emerged.

#### Willingness to interact

Fig. 3B and Table 2 show ratings for Willingness to Interact. The RM-ANOVA showed main effects for age, *F*(1,624) = 86.03, *p*≤.001, η_p_^2^ = .06, and for ethnicity, *F*(1,624) = 158.58, *p*≤.001, η_p_^2^ = .11. These main effects were subsumed by a significant interaction, *F*(1,624) = 154.40, *p*≤.001, η_p_^2^ = .11. Tukey HSD post-hoc analyses with Holm adjustment showed that Italian participants expressed the highest Willingness to Interact with pictures of Caucasian adults, and it differed statistically from their Willingness to Interact with Asian infants (*M* difference _(Caucasian Adults – Asian Infants)_ = .22, *SE* = .006, *p*≤.05), Caucasian infants (*M* difference _(Caucasian Adults – Caucasian Infants)_ = .21, *SE* = .003, *p*≤.05), and Asian adults (*M* difference _(Caucasian Adults – Asian Adults)_ = .71, *SE* = .005, *p*≤.001). The lowest Willingness to Interact Italian participants expressed was for Asian adults and that was significantly lower than for Asian infants (*M* difference _(Asian Adults – Asian Infants)_ = −.49, *SE* = .006, *p*≤.05) and Caucasian infants (*M* difference _(Asian Adults – Caucasian Infants)_ = −.50, *SE* = .005, *p*≤.05). No differences emerged for Willingness to Interact with infants (*M* difference _(Caucasian Infants – Asian Infants)_ = −.01, *SE* = .002, *ns*).

## Discussion

**Figure 4:**
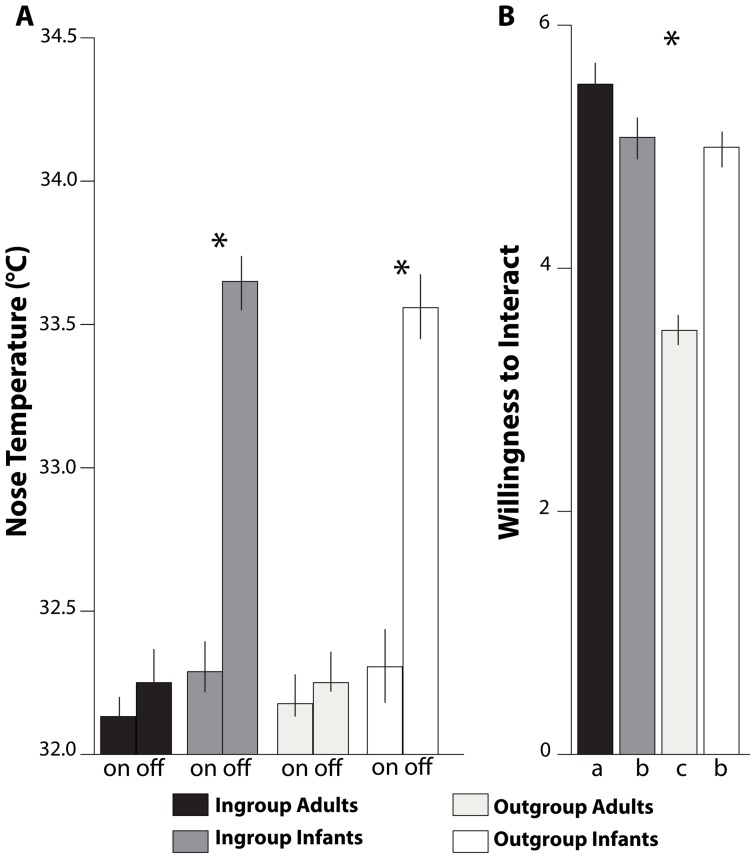
Japanese and Italian participants merged. Responses to the presentation of the faces of Ingroup adults (black), Ingroup infants (dark grey), Outgroup adults (light grey), and Outgroup infants (white). (A) Mean levels (± SEM) of the tip of the nose temperature at onset (on) and offset (off) of picture presentation. (B) Mean levels (± SEM) of scores for Willingness to Interact with the person. Bars with different subscripts (a, b, and c) are significantly different in Tukey HSD post-hoc tests.

In this study we tested physiological and behavioral reactions to infant and adult ingroup and outgroup faces in Japan and Italy. Fig. 4 combines and summarizes results of the two studies. All significant effects found in this study were medium-sized [39]. In accordance with our first hypothesis (H1), we found that both Japanese and Italian adults, although belonging to contrasting cultures, showed similar patterns of physiological and behavioral responses. These results also confirm our second hypothesis (H2), *viz*. both groups dissociate physiological and behavioral responses. The dissociation between physiological (emotional) and behavioral (judgmental) responsiveness underscores a distinction between automatic/autonomic and cognitive/cortical involvement.

In accord with our third hypothesis (H3), at the physiological level, both Japanese and Italians increased their facial temperatures in response to both ingroup and outgroup infant faces. By contrast, at the behavioral level, both Japanese and Italians expressed significant specific preferences for ingroup adults (H4). As shown in a number of behavioral studies for many types of stimuli, including faces, exposure tends to increase implicit ingroup attitudes [13] and attraction even when exposure is unconscious [14]–[16]. Our behavioral results accord with this view. Specifically, we found that faces of people in the same race and age group elicit from adults greater willingness to interact than faces from another race or age group. These preferences are evident at a behavioral level and may be associated with higher-order cognitive processing. Taken together, our results suggest that adults' automatic emotional responses to infant stimuli appear to be mediated by subcortical structures (i.e., autonomic nervous system) and are not dependent on direct caregiving exposure, whereas adults' behavioral cognitive responses are mediated by higher-order processing based on social acceptance and cultural exposure.

Facial temperature change is related to different arousal levels and actions. Perhaps one or both of two mechanisms may be involved. First, Sprengelmeyer et al. [40] implicated the likely involvement of the hormones estrogen and progestogen which alter physiological responses to cute baby faces. They suggested that neural substrates involved in reward processing and maternal behavior are possible candidate structures linked to these processes [41]. It is also possible that physiological response to an infant face is comparable to that of the orienting response, which is characterized by vasodilation of the face as blood is distributed from the extremities towards the head. The orienting response is also regulated by the autonomic nervous system [42], and the response tends to weaken if elicited continually because of habituation. However, it has been proposed that orienting responses triggered by stimuli with high emotional valence (and we believe that infant faces have high emotional valence for evolutionary reasons) do not habituate [43]. Williams and colleagues [44] used integration of fMRI and skin conductance to show how stimuli with higher orienting salience are processed in the ventromedial prefrontal cortex (a region associated with encoding emotional valence [45]) and in the anterior cingulate cortex (which controls a wide variety of autonomic functions, such as regulating blood pressure and heart rate). The emotional significance of a stimulus (here infant faces) can affect the intensity of the orienting response in focusing attention on a subject. The increase of facial temperature measured via infrared thermography supports *kindchenschema* theory and accords with other studies using different technologies (e.g., MEG [3]–[4], [11]; fMRI [7]; IAT [12]) that point to a fast acting, automatic, unconscious response that directs attention to the infant face and is associated with positive emotions.

Before concluding, acknowledging some limitations of these experiments suggests future directions of research. In neither group for either measure did females show an ingroup preference for the infant faces. This result may be due to the fact that our sample (young non-mother females) did not have direct parenting experience with young infants. Indeed, it may be possible that testing mothers would show preferences for ingroup infant faces due to their greater exposure to ingroup infant faces. We compared only two cultures and found no differences in the pattern of results in Japanese and Italian participants. Although these results test the hypothesis that physiological emotional responses to infant faces might be widely generalizable, it would be desirable to extend this design to other cultural contexts, for example ones where solicitousness toward babies is not a given [46] or where there are even distributions of ingroup and outgroup people in the society (e.g., European Americans and Latin Americans in Miami, FL). A related limitation of this work is the difficulty of assessing level of participants' exposure to infant versus adult faces. We assumed that people from a single cultural context are mainly exposed to faces, images, and symbols representative of their context. However, because of globalization and mass media, even people of one group might be exposed to faces, images, and symbols of other (even distant) groups. For this reason, future studies should seek new ways of assessing level of stimulus exposure. In this connection, however, it is reasonable to note that exposure to outgroup faces would work against our hypothesis. Another limitation to consider is the accuracy of the thermal images. The thermal imaging system we used (Avio TVS-200EX) allows recording with a maximum speed of 1 frame per 3 s. Moreover, the captured frame has a resolution of 60 fps (60 Hz), which provides an accurate measure for each time-point. However, this field of investigation of change in human face temperature is still novel, so further testing and new devices may refine and yield images with better resolution. Finally, at this stage it appears that changes of skin temperature are generally associated with greater arousal or activation; however, it is not possible to discern the psychological meaning of that activation. Consequently, future studies might undertake multiple assessments (i.e., heart rate, galvanic skin response) and model psychological meaning of these physiological responses.

Here, we detected a dissociation between physiological and behavioral responses to infant and adult faces in culturally diverse participants. Japanese and Italians showed similar activation toward ingroup and outgroup infant faces at a physiological level, but each expressed significant preference for adult ingroup faces at a behavioral level. These results expose the multilevel nature of human interaction, where physiological and behavioral systems function to moderate social relationships.
